# Design of a High-Sensitivity Microstrip Patch Sensor Antenna Loaded with a Defected Ground Structure Based on a Complementary Split Ring Resonator

**DOI:** 10.3390/s20247064

**Published:** 2020-12-10

**Authors:** Junho Yeo, Jong-Ig Lee

**Affiliations:** 1 School of ICT Convergence, Daegu University, 201 Daegudae-ro, Gyeongsan 38453, Korea; 2 Department of Applied Electronics Engineering, Dongseo University, San69-1, Jurye-2dong, Sasang-gu, Busan 47011, Korea; leeji@gdsu.dongseo.ac.kr

**Keywords:** defected ground structure, double-ring complementary split ring resonator, radiating edge, microstrip patch sensor antenna, permittivity characterization

## Abstract

A comparative study to determine the most highly sensitive resonant frequency among the first four resonant frequencies of a conventional patch antenna and defected ground structure (DGS)-loaded patch antennas using commonly used DGS geometries in the literature, such as a rectangular slit, single-ring complementary split ring resonators (CSRRs) with different split positions, and double-ring CSRRs (DR-CSRRs) with different locations below the patch, for relative permittivity measurement of planar materials was conducted. The sensitivity performance for placing the DGS on two different locations, a center and a radiating edge of the patch, was also compared. Finally, the effect of scaling down the patch size of the DGS-loaded patch antenna was investigated in order to enhance the sensitivities of the higher order resonant frequencies. It was found that the second resonant frequency of the DR-CSRR DGS-loaded patch antenna aligned on a radiating edge with a half scaled-down patch size shows the highest sensitivity when varying the relative permittivity of the material under test from 1 to 10. In order to validate the simulated performance of the proposed antenna, the conventional and the proposed patch antennas were fabricated on 0.76-mm-thick RF-35 substrate, and they were used to measure their sensitivity when several standard dielectric substrate samples with dielectric constants ranging from 2.17 to 10.2 were loaded. The measured sensitivity of the second resonant frequency for the proposed DGS-loaded patch antenna was 4.91 to 7.72 times higher than the first resonant frequency of the conventional patch antenna, and the measured performance is also slightly better compared to the patch antenna loaded with a meander-line slot on the patch.

## 1. Introduction

Precise characterization of the dielectric constant or the relative permittivity of a material is very important in various applications, such as material science, wireless communications, agriculture, chemistry, and the biomedical, healthcare, and food industries [1]. Microwave technology–based measurement methods have been extensively studied and widely used, and permittivity measurement can be divided as non-resonant and resonant. Non-resonant methods derive the material’s permittivity from changes in the characteristic impedance and wave velocity of electromagnetic waves, measuring reflection and transmission characteristics [2,3,4,5]. Resonant methods determine permittivity from the shift in resonant frequencies of a resonant structure [6,7,8,9].

Recently, resonant methods with planar resonators on planar transmission lines (for example, by using split ring resonator (SRR) and complementary SRR (CSRR) structures) have become popular because they are compact in size with a simple geometry, are ease to fabricate, and carry a low cost [10,11,12,13,14,15,16,17,18,19,20,21,22,23]. In fact, CSRR structures inserted on the ground plane of planar transmission lines in the form of a single ring or a double ring can be considered a defected ground structure (DGS) [24]. The DGS is known as a resonant slot etched as a single defect or in periodic configuration with a small period number on the ground plane, and its concept has evolved from the studies of photonic band gap structures as a simplified version with similar properties [25]. Two important characteristics of the DGS are a slow wave effect in the pass band and a band stop property [26]. The DGS has been widely used for various microwave filter designs such a low pass filter, a band stop filter, and a band pass filter [27]. The DGS geometries reported in the literature include rectangular dumbbell, circular dumbbell, U-shaped, V-shaped, H-shaped, cross-shaped, spiral, concentric ring, CSRR, and fractal [25]. The DGS has also been used to improve the efficiency of planar microwave amplifiers. For antenna applications, the DGS has been used to enhance various properties such as miniaturization, cross polarization reduction, mutual coupling reduction in antenna arrays, notch band generation, multi-band operation, and higher order harmonic suppression [27]. A dual-band microstrip patch antenna with two double-ring CSRRs (DR-CSRRs) on the ground plane near the inset of the patch was proposed to resonate at around 3.87 GHz and 5.46 GHz [28]. The first operating band is contributed by the DR-CSRRs, whereas the second is originated by the patch. A microstrip patch antenna with two by two DR-CSRRs etched on the ground plane was introduced for size reduction and gain enhancement [29]. The measured bandwidth was 100 MHz from 2.4 GHz to 2.5 GHz with a gain of 5.93 dBi at around the center frequency. A reduced– size microstrip circular patch antenna from etching a single circular DR-CSRR on the ground plane resonating at 6.11 GHz was reported [30]. The effective footprint of the antenna was reduced by nearly 64% compared to the conventional patch antenna. However, the permittivity sensitivities of the multiple resonant frequencies generated by double-ring or single-ring CSRRs on the ground plane of the microstrip patch antenna when a material under test (MUT) is placed below the ground plane have not been systematically investigated in the literature.

In this paper, a DGS-loaded high-sensitivity microstrip patch sensor antenna (MPSA) based on a DR-CSRR is proposed for permittivity characterization. A conventional patch antenna and MPSAs loaded with a rectangular slit (RS) or single-ring CSRRs (SR-CSRRs) with different split positions on the ground plane were used to compare the sensitivities of the resonant frequencies of the proposed DR-CSRR DGS-loaded MPSA. The shift in the resonant frequency of the input reflection coefficient (S_11_) has been measured when placing the planar dielectric MUT above the patch, for the conventional antenna. Conversely, the MUT was placed below the ground plane for the DGS-loaded MPSAs. In order to find the highest sensitive resonant frequency, the sensitivities of the first four resonant frequencies of each MPSA were calculated and compared when the relative permittivity of the MUT (with a thickness of 1.6 mm) was varied from 1 to 10. Full-wave simulations were performed using CST Microwave Studio.

## 2. Design of DGS-loaded MPSAs

**Figure 1:**
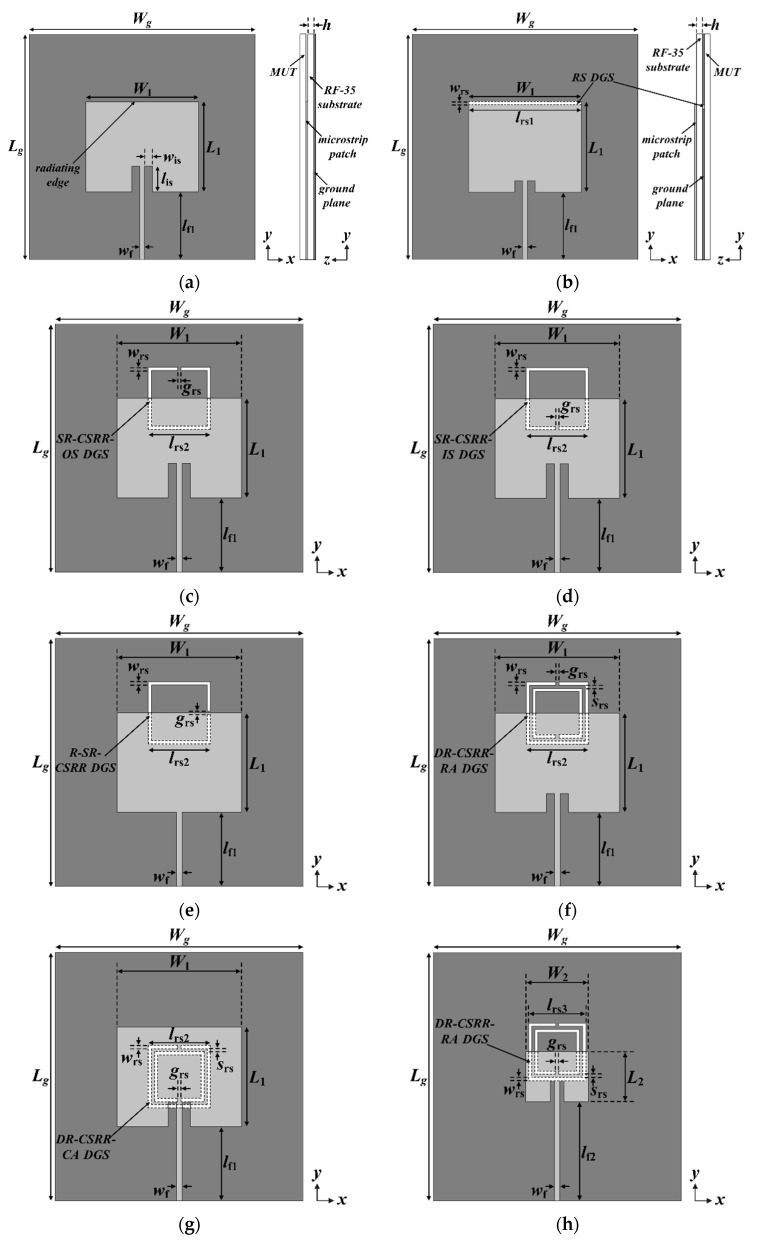
Geometries of the microstrip patch sensor antennas (MPSAs): (**a**) a conventional MPSA without a defected ground structure (DGS); (**b**) an rectangular slit (RS) DGS-loaded MPSA; (**c**) an SR-CSRR outside split (SR-CSRR-OS) DGS-loaded MPSA; (**d**) an SR-CSRR inside split (SR-CSRR-IS) DGS-loaded MPSA; (**e**) a 90-degree rotated SR-CSRR (R-SR-CSRR) DGS-loaded MPSA; (**f**) a DR-CSRR radiating edge aligned (DR-CSRR-RA) DGS-loaded MPSA; (**g**) a DR-CSRR center aligned (DR-CSRR-CA) DGS-loaded MPSA; and (**h**) the proposed scaled DR-CSRR-RA DGS-loaded MPSA.

**Figure 2:**
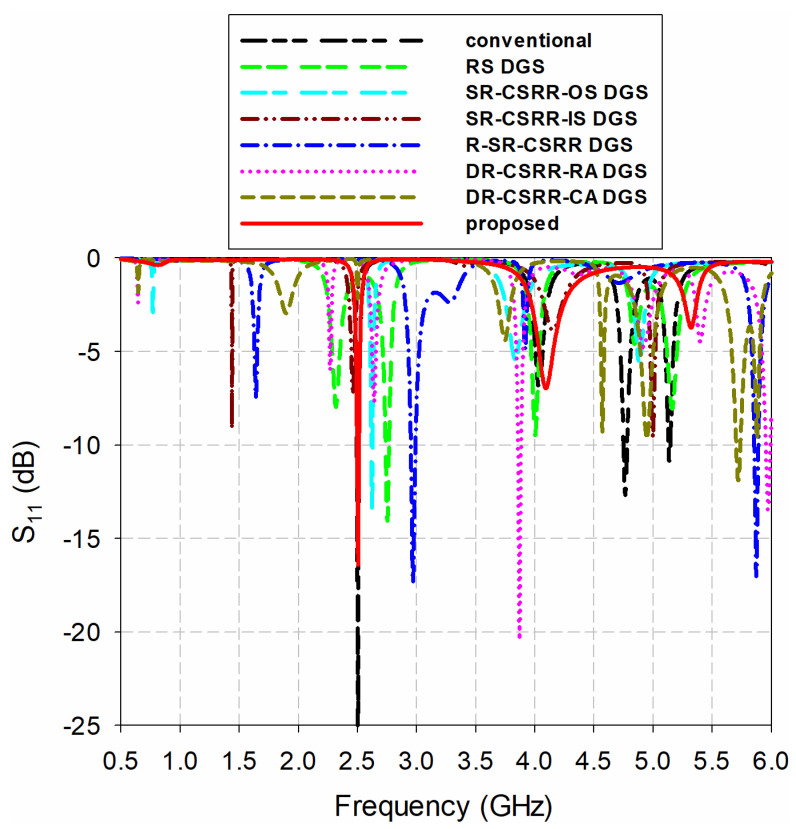
Input reflection coefficient characteristics vs. frequency for the eight MPSAs in Figure 1.

Figure 1 shows the geometries of a conventional inset-fed rectangular patch antenna, an RS DGS-loaded MPSA, an SR-CSRR outside split (SR-CSRR-OS) DGS-loaded MPSA, an SR-CSRR inside split (SR-CSRR-IS) DGS-loaded MPSA, a 90-degree rotated SR-CSRR (R-SR-CSRR) DGS-loaded MPSA, a DR-CSRR radiating edge aligned (DR-CSRR-RA) DGS-loaded MPSA, a DR-CSRR center aligned (DR-CSRR-CA) DGS-loaded MPSA, and the proposed scaled DR-CSRR-RA DGS-loaded MPSA. The corresponding S_11_ characteristics of the eight MPSAs under unloaded conditions are shown in Figure 2. An RF-35 substrate (*ε*_r_ = 3.5, tan *δ* = 0.0018, *h* = 0.76 mm) was used to design the MPSAs.

First, the conventional inset-fed rectangular patch antenna was designed to have its first resonant frequency at *f*_r1_ = 2.5 GHz under unloaded conditions by using equations from [31].

The width and length of the rectangular patch obtained by using equations were *W*_1_ = 40.0 mm and *L*_1_ = 31.9 mm, while those of the 50-ohm microstrip feed line were *w*_f_ = 1.66 mm and *l*_f1_ = 24.5 mm. Those of the inset were *w*_is_ = 2.8 mm and *l*_is_ = 9 mm, and both width and length of the ground plane were the same: *W*_g_ = *L*_g_ = 80 mm. The MUT was placed as a superstrate (above the patch) for loaded conditions, as seen in Figure 1a. There were three other higher order resonant frequencies: *f*_r2_ = 4.046 GHz, *f*_r3_ = 4.766 GHz, and *f*_r4_ = 5.138 GHz.

The RS DGS was etched on the ground plane of the conventional patch antenna along the radiating edge opposite the microstrip feed line. (See Figure 1b) Length and width of the RS DGS were *l*_rs1_ = 40.0 mm and *w*_rs_ = 1 mm, respectively. The RS DGS location was selected for two main reasons: The electric field concentration is highest along the radiating edge of the patch, as is the case for permittivity sensitivity related to the capacitive perturbation from the electric field as it interacts with the dielectric MUT, are highest at this position. We reduced the length of the inset to enhance the impedance matching. The first four resonant frequencies were *f*_r1_ = 2.316 GHz, *f*_r2_ = 2.75 GHz, *f*_r3_ = 4.002 GHz, and *f*_r4_ = 4.844 GHz.

Thirdly, an SR-CSRR-OS DGS centered on the radiating edge of the patch was inserted on the ground plane of the conventional patch antenna, as shown in Figure 1c. The location of the split point is outside the patch. The dimensions of the SR-CSRR-OS DGS are as follows: *l*_rs2_ = 20.0 mm, *w*_rs_ = 1 mm, and *g*_rs_ = 1 mm. In this case, the first four resonant frequencies appeared at *f*_r1_ = 0.768 GHz, *f*_r2_ = 2.62 GHz, *f*_r3_ = 3.836 GHz, and *f*_r4_ = 4.886 GHz.

Fourth, an SR-CSRR-IS DGS with the split point located inside the patch was added on the ground plane of the conventional patch antenna, as shown in Figure 1d. The dimensions of the SR-CSRR-IS are the same as the SR-CSRR-OS DGS. The first four resonant frequencies were at *f*_r1_ = 1.436 GHz, *f*_r2_ = 2.458 GHz, *f*_r3_ = 3.406 GHz, and *f*_r4_ = 4.152 GHz.

Fifth, an R-SR-CSRR DGS was etched on the ground plane of the conventional patch antenna, as shown in Figure 1e. The SR-CSRR-OS DGS was rotated 90 degrees clockwise. For this, the inset was removed in order to enhance the impedance matching, and the first four resonant frequencies were at *f*_r1_ = 1.638 GHz, *f*_r2_ = 2.968 GHz, *f*_r3_ = 3.28 GHz, and *f*_r4_ = 3.918 GHz.

Sixth, a DR-CSRR-RA DGS centered on the radiating edge of the patch was added on the ground plane of the conventional patch antenna, as shown in Figure 1f. The split point of the outer ring is located outside the patch, whereas that of the inner ring is inside the patch. The dimensions of the DR-CSRR-RA DGS are as follows: *l*_rs2_ = 20.0 mm, *w*_rs_ = 1 mm, *g*_rs_ = 1 mm, and *s*_rs_ = 1 mm. The first four resonant frequencies appeared at *f*_r1_ = 0.64 GHz, *f*_r2_ = 2.266 GHz, *f*_r3_ = 2.638 GHz, and *f*_r4_ = 3.87 GHz.

Seventh, a DR-CSRR-CA DGS centered on the center of the patch was added on the ground plane of the conventional patch antenna, as shown in Figure 1g, in order to compare the sensitivities of the resonant frequencies, whether the location of the DR-CSRR is centered on the radiating edge or on the patch center. The dimensions of the DR-CSRR-CA DGS are the same as those of the DR-CSRR-OS DGS. The first four resonant frequencies were at *f*_r1_ = 0.644 GHz, *f*_r2_ = 1.896 GHz, *f*_r3_ = 2.498 GHz, and *f*_r4_ = 3.746 GHz.

Finally, the proposed scaled DR-CSRR-RA DGS was etched on the ground plane of a scaled patch antenna, as shown in Figure 1h, in order to improve the sensitivities of the resonant frequencies. The dimensions of the patch were reduced to half: *W*_2_ = 20.0 mm and *L*_2_ = 16.0 mm. The width and length of the inset were modified to *w*_is2_ = 1.4 mm and *l*_is2_ = 7 mm, respectively, whereas the length of the 50-ohm microstrip feed line was increased to *l*_f2_ = 32.0 mm. The outer length of the DR-CSRR-RA DGS was slightly reduced to *l*_rs3_ = 18.5 mm, and the other parameters remained the same. The first four resonant frequencies appeared at *f*_r1_ = 0.808 GHz, *f*_r2_ = 2.5 GHz, *f*_r3_ = 4.09 GHz, and *f*_r4_ = 5.318 GHz.

## 3. Sensitivity Comparison of First Four Resonant Frequencies

This section compares the first four resonant frequencies for the eight. We compare the sensitivities by measuring the shift in the resonant frequencies of the input reflection coefficient. As mentioned before, the MUT was placed as a superstrate (above the patch) for the conventional MPSA, but below the ground plane for the DGS-loaded MPSAs.

**Figure 3:**
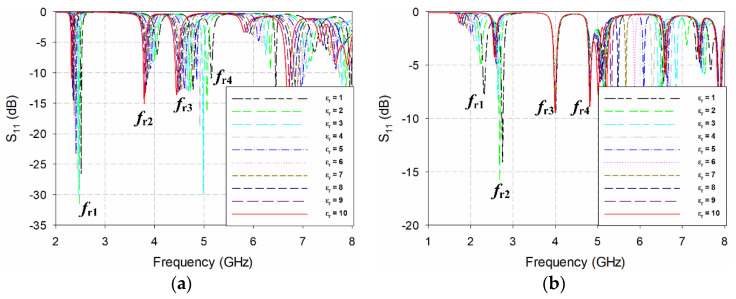

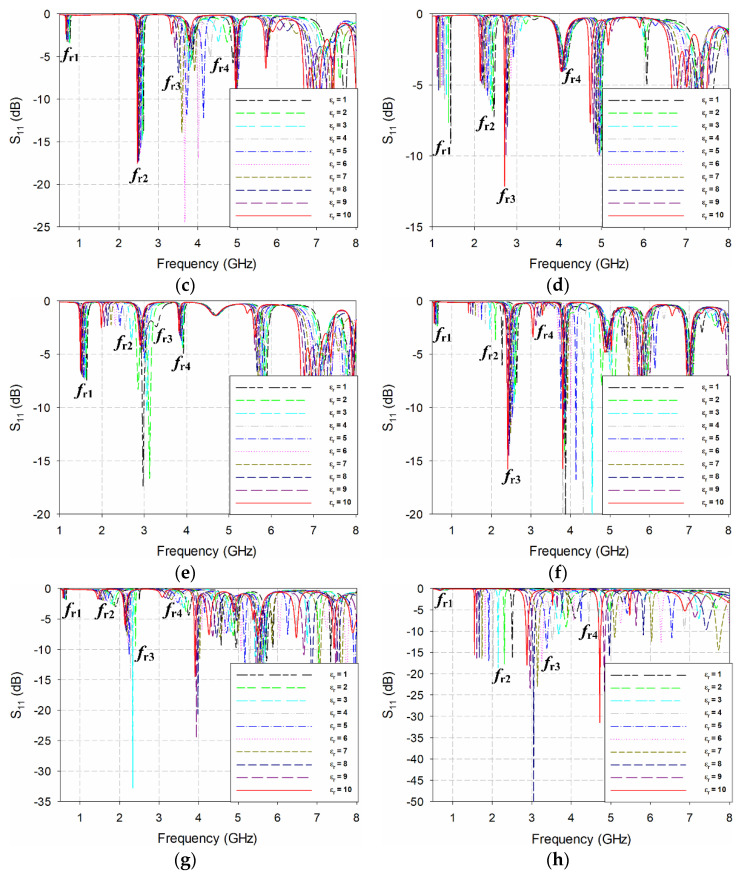
S_11_ characteristics when varying the relative permittivity of the MUT from 1 to 10 for: (**a**) the conventional MPSA without a DGS; (**b**) the RS DGS-loaded MPSA; (**c**) the SR-CSRR-OS DGS-loaded MPSA; (**d**) the SR-CSRR-IS DGS-loaded MPSA; (**e**) the R-SR-CSRR DGS-loaded MPSA; (**f**) the DR-CSRR-RA DGS-loaded MPSA; (**g**) the DR-CSRR-CA DGS-loaded MPSA; and (**h**) the proposed scaled DR-CSRR DGS-loaded MPSA.

**Figure 4:**
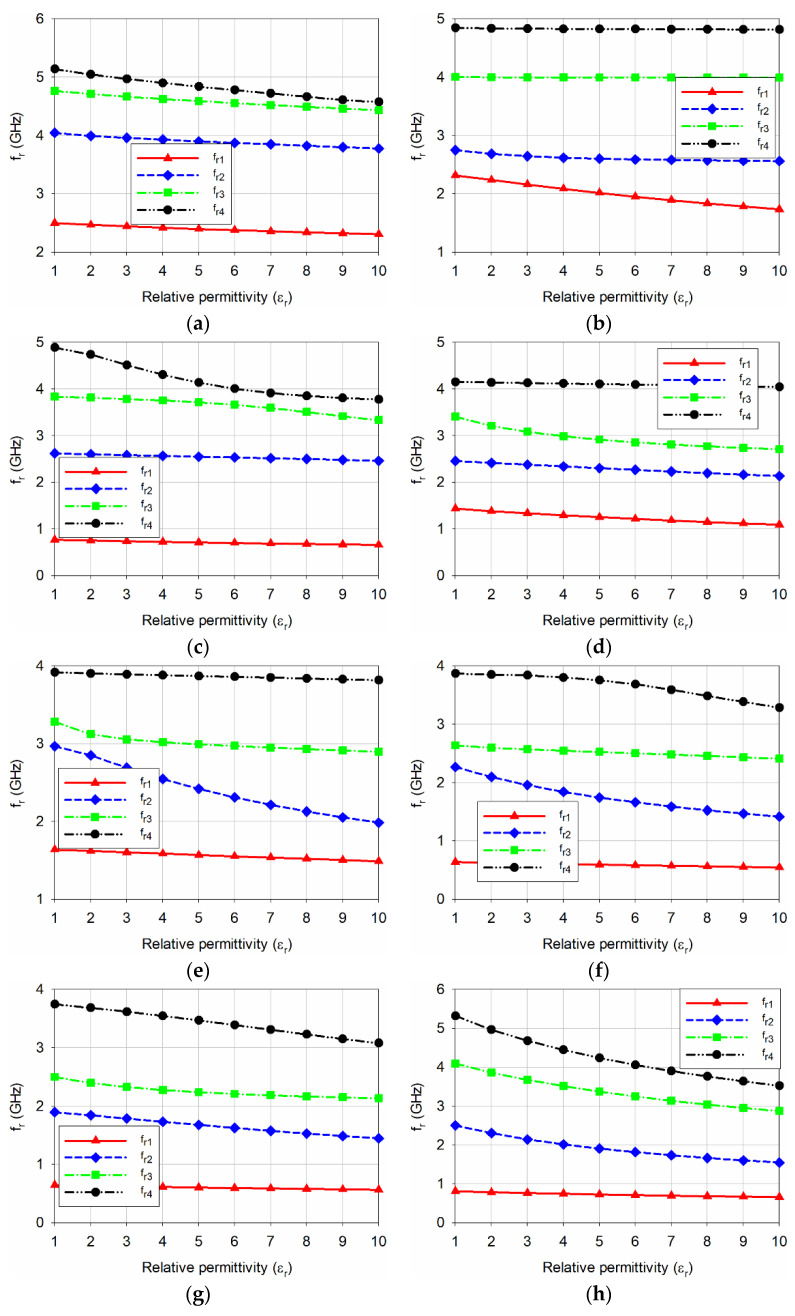
Variations on the first four resonant frequencies when varying the relative permittivity of the MUT from 1 to 10 for: (**a**) the conventional MPSA without DGS; (**b**) the RS DGS-loaded MPSA; (**c**) the SR-CSRR-OS DGS-loaded MPSA; (**d**) the SR-CSRR-IS DGS-loaded MPSA; (**e**) the R-SR-CSRR DGS-loaded MPSA; (**f**) the DR-CSRR-RA DGS-loaded MPSA; (**g**) the DR-CSRR-CA DGS-loaded MPSA; and (**h**) the proposed scaled DR-CSRR DGS-loaded MPSA.

The S_11_ characteristics of the eight MPSAs in Figure 1 are shown in Figure 3. We varied the relative permittivity (*ε*_r_) of the MUT from 1 to 10 (in increments of 1), with a zero-loss tangent. The width and length of the MUT were the same as those of the ground plane, and the thickness chosen was 1.6 mm. That is because the thickest substrates available from Taconic Inc., New York, NY, USA is around 1.6 mm. The characteristics of the first four resonant frequencies for the eight MPSAs, as extracted from Figure 3, are shown in Figure 4. Table 1 summarizes their values.

**Figure 5:**
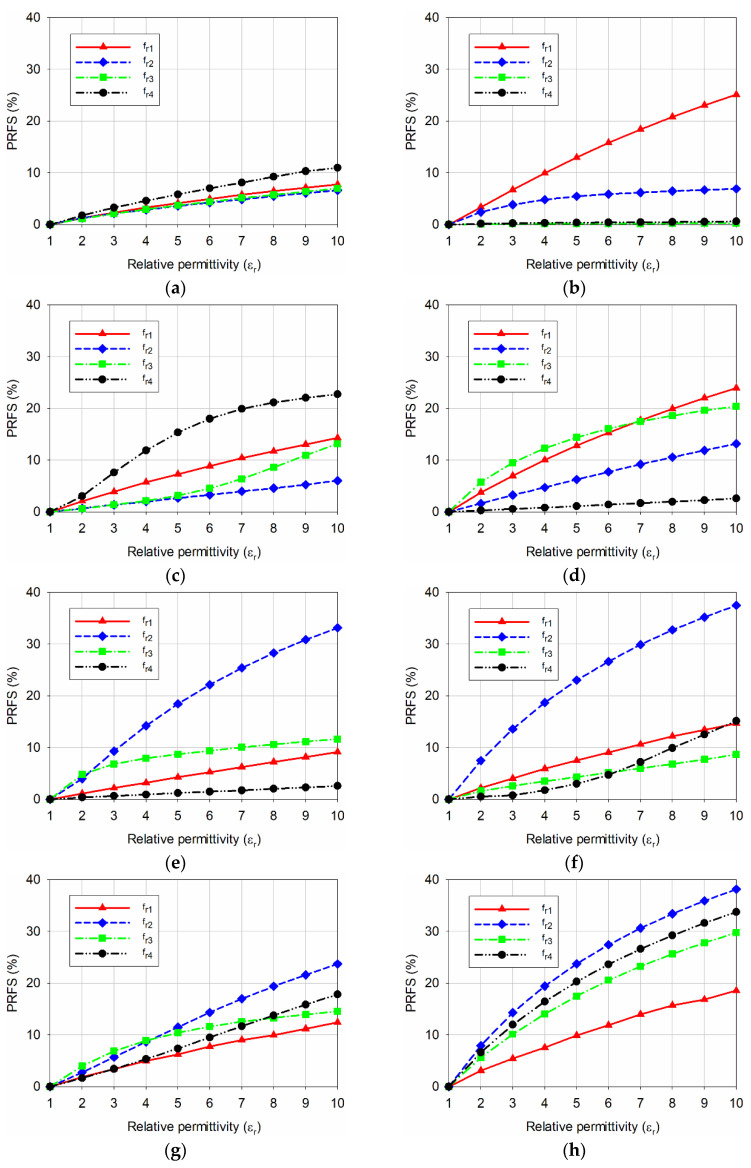
PRFS of the first four resonant frequencies when varying the relative permittivity of the MUT from 1 to 10 for: (**a**) the conventional MPSA without DGS; (**b**) the RS DGS-loaded MPSA; (**c**) the SR-CSRR-OS DGS-loaded MPSA; (**d**) the SR-CSRR-IS DGS-loaded MPSA; (**e**) the R-SR-CSRR DGS-loaded MPSA; (**f**) the DR-CSRR-RA DGS-loaded MPSA; (**g**) the DR-CSRR-CA DGS-loaded MPSA; and (**h**) the proposed scaled DR-CSRR DGS-loaded MPSA.

To compare the sensitivity of the eight MPSAs, the percent relative frequency shift (PRFS) of the first four resonant frequencies for the eight MPSAs, was calculated by using Table 1, and the results are shown in Figure 5. PRFS is defined as a percentage of the ratio of the difference between the resonant frequencies for unloaded and loaded conditions to the resonant frequencies for unloaded conditions [32,33], as shown in Equation (1).

(1) $$PRFS=\frac{{\Delta f}_{r}}{f_{r}}=\frac{f_{ru}-f_{rl}}{f_{ru}}\times 100(\%)$$

(2) $$PRFSE=\frac{{PRFS}_{proposed}}{{PRFS}_{conventional}}$$

where Δ*f*_r_ is the shift in the resonant frequencies of the eight MPSAs, *f*_ru_ is the resonant frequencies of the eight MPSAs for unloaded conditions, *f*_rl_ is the resonant frequencies of the eight MPSAs for loaded conditions.

For the conventional MPSA, the PRFS of the first four resonant frequencies was less than 11.0% when the relative permittivity of the MUT increased from 1 to 10, and the PRFS of *f*_r4_ was the highest among the four. For the RS DGS-loaded MPSA, the PRFS of *f*_r1_ increased to 25.1%, but PRFS for the other resonant frequencies was less than 6.9%. For the SR-CSRR-OS DGS-loaded MPSA, the PRFS of *f*_r4_ increased to 22.8%, but PRFS for the other resonant frequencies was less than 14.3%. For the SR-CSRR-IS DGS-loaded MPSA, PRFS for *f*_r1_ and *f*_r3_ were 24.0% and 20.4%, respectively, but PRFS for the other resonant frequencies was less than 13.2%. For the R-SR-CSRR DGS-loaded MPSA, the PRFS of *f*_r4_ increased to 33.2%, but PRFS for all other resonant frequencies was less than 11.6%. For the DR-CSRR-RA DGS-loaded MPSA, the PRFS of *f*_r2_ increased to 37.5%, but PRFS for the other resonant frequencies was less than 15.1%. For the DR-CSRR-CA DGS-loaded MPSA, the PRFS of *f*_r2_ increased to 23.7%, but the PRFS for the other resonant frequencies was less than 17.8%.

Hence, when sensitivities are compared using the PRFS of the highest sensitive resonant frequency, the PRFS of *f*_r2_ for the DR-CSRR-RA DGS-loaded MPSA was better than the DR-CSRR-CA DGS-loaded MPSA, and the radiating edge of the patch might be a better location in order to achieve higher sensitivity. Finally, for the proposed scaled DR-CSRR-RA DGS-loaded MPSA, the PRFS for *f*_r2_, *f*_r3_, and *f*_r4_ increased to 38.2%, 29.8%, and 33.8%, respectively, and the PRFS for *f*_r1_ increased to 19.6%. Although the PRFS of *f*_r1_ and *f*_r2_ for the proposed scaled DR-CSRR-RA DGS-loaded MPSA increased a little compared to the original DR-CSRR-RA DGS-loaded MPSA in Figure 1f, the PRFS for *f*_r3_ and *f*_r4_ was considerably enhanced by more than two times. Therefore, *f*_r2_, *f*_r3_, and *f*_r4_ of the proposed scaled DR-CSRR-RA DGS-loaded MPSA might be used for differential sensing with similar sensitivity, or for a multi-band microwave sensor with a single resonating element.

As described in Equation (2), PRFSE is defined as the enhancement in the PRFS of the resonant frequencies of the proposed MPSA, compared to the conventional MPSA. According to [32], PRFSE is almost the same as sensitivity enhancement, and can be used as a measure of sensitivity enhancement. For example, when the permittivity of the MUT was *ε*_r_ = 2, the PRFS of *f*_r1_ for the conventional MPSA was 1.3%, and PRFS for *f*_r2_, *f*_r3_, and *f*_r4_ of the proposed scaled DR-CSRR-RA DGS-loaded MPSA were 7.9%, 5.6%, and 6.6%, respectively. Therefore, PRFSE for *f*_r2_, *f*_r3_, and *f*_r4_ of the proposed scaled DR-CSRR-RA DGS-loaded MPSA were 6.08, 4.31, and 5.08, respectively, compared to *f*_r1_ of the conventional MPSA.

As the permittivity of the MUT increased to *ε*_r_ = 10, the PRFS of *f*_r1_ for the conventional MPSA was 7.8%, and PRFS for *f*_r2_, *f*_r3_, and *f*_r4_ of the proposed scaled DR-CSRR-RA DGS-loaded MPSA were 38.2%, 29.9%, and 33.8%, respectively. Therefore, PRFSE for *f*_r2_, *f*_r3_, and *f*_r4_ for the proposed scaled DR-CSRR-RA DGS-loaded MPSA were 4.90, 3.83, and 4.33, respectively.

**Figure 6:**
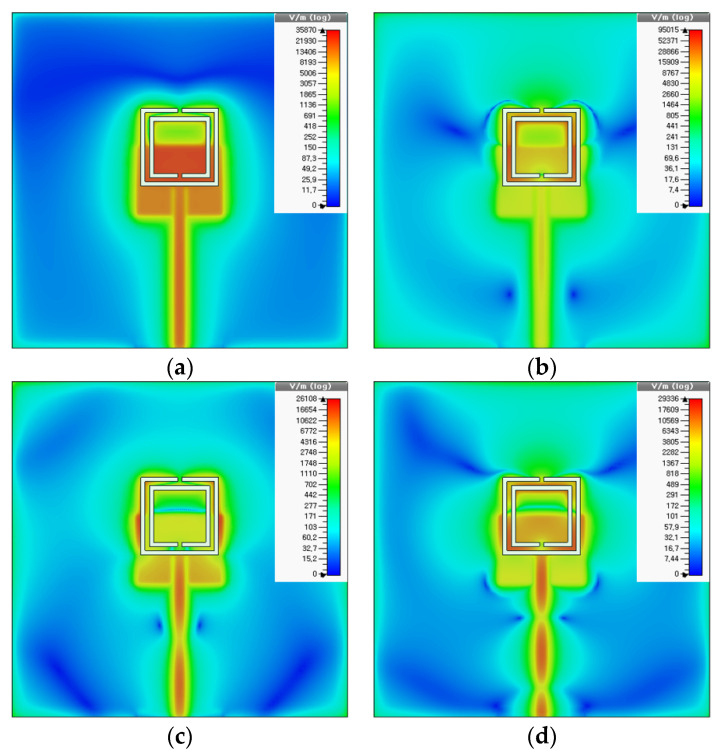
Electric field distributions on the ground plane at the first four resonant frequencies of the proposed scaled DR-CSRR-RA DGS-loaded MPSA: (**a**) 0.808 GHz; (**b**) 2.5 GHz; (**c**) 4.09 GHz; and (**d**) 5.318 GHz.

Figure 6 shows the electric field distributions of the first four resonant frequencies on the ground plane for the proposed scaled DR-CSRR-RA DGS-loaded MPSA at 0.808 GHz, 2.5 GHz, 4.09 GHz, and 5.318 GHz. At the first resonant frequency, the electric field distribution was widespread on the area below the patch. For the second resonant frequency, it was concentrated on the area between the outer and inner rings, and the magnitude of the electric field was the highest among the four. At the third resonant frequency, the electric field was distributed on part of the outer ring, whereas it was concentrated on part of the area between the outer and inner rings for the fourth resonant frequency.

**Figure 7:**
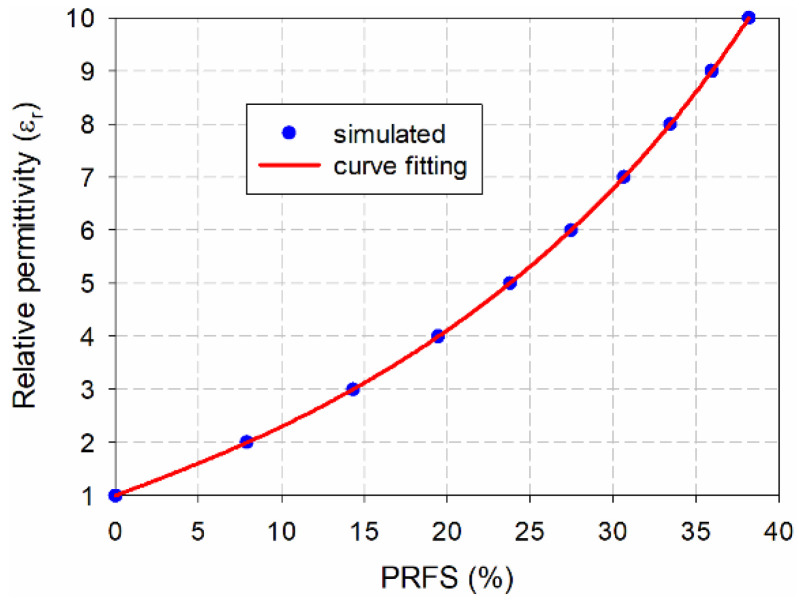
Simulated and curve-fitted relative permittivity of the material under test (MUT) vs. percent relative frequency shift (PRFS).

For the proposed scaled DR-CSRR-RA DGS-loaded MPSA, to determine the relationship between the PRFS of the second resonant frequency and the relative permittivity of the MUT, we used a curve-fitting tool for SigmaPlot, by Systat Software Inc., and choose a fifth-order polynomial function for the fitting function. When the MUT’s relative permittivity (*ε*_r_) was varied, the PRFS of the simulated second resonant frequency for the proposed scaled DR-CSRR-RA DGS-loaded MPSA was used to derive the Equation (3). The simulated and curve-fitted relative permittivities of the MUT as a function of the PRFS are compared in Figure 7.

(3) $$\begin{array}{lll} \varepsilon_{r} & = & 1.0001+0.1170\times PRFS+4.2428\times 10^{-4}\times {PRFS}^{2}+1.0654\times 10^{-4}\times {PRFS}^{3}-2.1748\times 10^{-6}\times {PRFS}^{4}\\ & + & 3.2260\times 10^{-8}\times {PRFS}^{5} \end{array}$$

**Figure 8:**
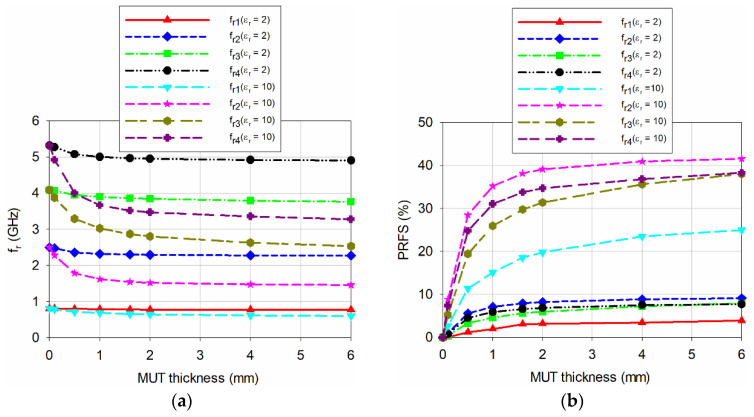
Variations on the first four resonant frequencies for the proposed scaled DR-CSRR DGS-loaded MPSA when varying the thickness of the MUT: (**a**) *f*_r_; and (**b**) PRFS.

Next, the effect of varying the thickness of the MUT from 0.1 to 6 mm on the first four resonant frequencies for the proposed scaled DR-CSRR-RA DGS-loaded MPSA was investigated for *ε*_r_ = 2 and 10, as shown in Figure 8. As the thickness of the MUT increased, the resonant frequencies decreased and their PRFS increased with nonlinear behaviors, and the amount of decrease or increase was saturated. In addition, the most sensitive resonant frequency in terms of PRFS for varying the thickness of the MUT was the second resonant frequency.

**Figure 9:**
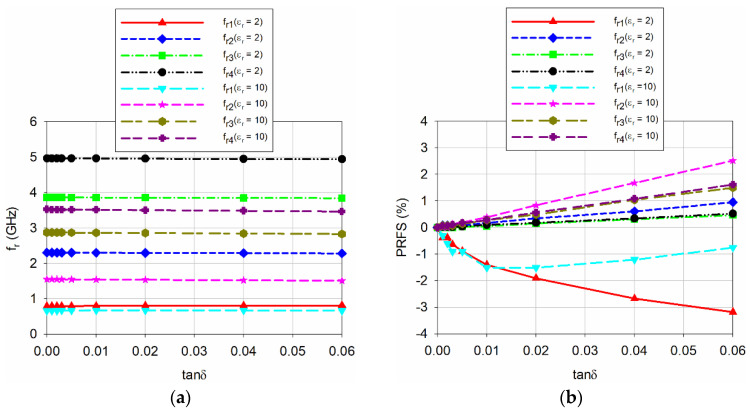
Variations on the first four resonant frequencies for the proposed scaled DR-CSRR DGS-loaded MPSA when varying the loss tangent of the MUT: (**a**) *f*_r_; and (**b**) PRFS.

Figure 9 shows the effect of varying the loss tangent of the MUT from 0.001 to 0.06 on the first four resonant frequencies for the proposed scaled DR-CSRR-RA DGS-loaded MPSA with the thickness of the MUT fixed at 1.6 mm. 9 different values of the loss tangent were used for simulation; 0, 0.001, 0.002, 0.003, 0.005, 0.01, 0.02, 0.04, 0.06. The relative permittivity of the MUT was considered for two cases: *ε*_r_ = 2 and 10. When the loss tangent increased, the resonant frequencies decreased and their PRFS increased linearly for the second, third, and fourth resonant frequencies. However, the variations for the first resonant frequency were opposite compared to the second, third, and fourth resonant frequencies. The first resonant frequency moved toward high frequency nonlinearly, and its PRFS increased with minus values. For *ε*_r_ = 10, both the resonant frequency and PRFS decreased slightly. The most sensitive resonant frequency in terms of PRFS among three higher order resonant frequencies was the second resonant frequency. When the relative permittivity of the MUT was as low as *ε*_r_ = 2, PRFS of the second resonant frequency increased about 0.96% when tan *δ* varied from 0 to 0.06. As *ε*_r_ increased to 10, PRFS increased about 2.52%. If we limit the loss tangent of the MUT less than 0.003 for low-loss cases, the variation in PRFS ranged from 0.09% to 0.13%. Therefore, the effect of the loss tangent on the resonant frequencies would be relatively insignificant for low loss MUTs.

## 4. Experiment Results and Discussion

**Figure 10:**
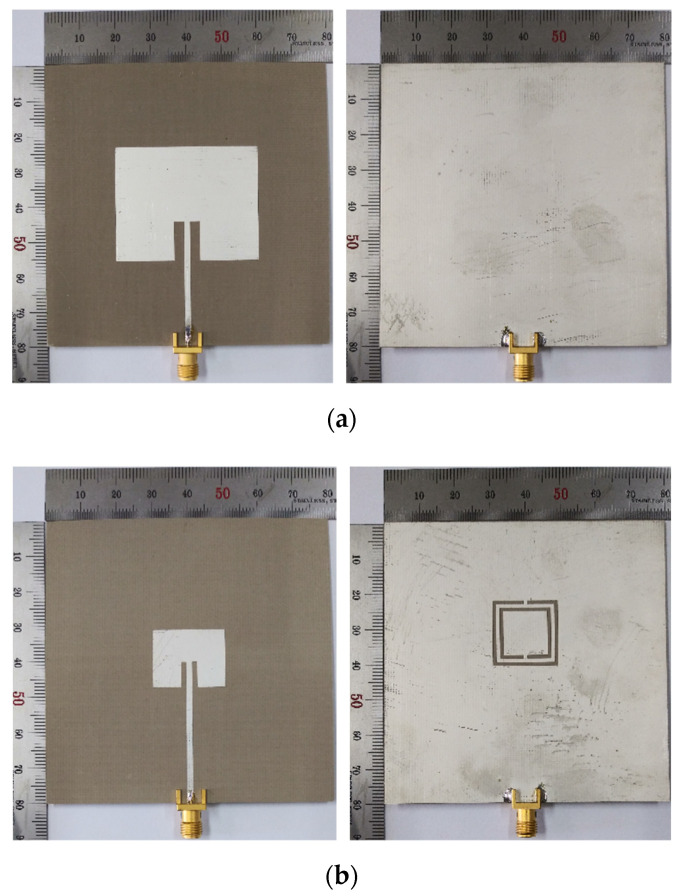
Photographs of the fabricated MPSAs: (**a**) the conventional MPSA; and (**b**) the proposed scaled DR-CSRR-RA DGS-loaded MPSA.

**Figure 11:**
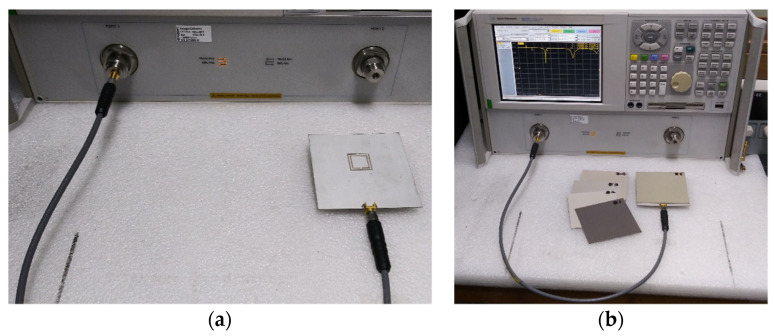
Experiment setups: (**a**) unloaded; (**b**) loaded.

The prototypes of the conventional and the proposed scaled DR-CSRR-RA DGS-loaded MPSAs are shown in Figure 10, as fabricated on an RF-35 substrate, where *ε*_r_ = 3.5, tan *δ* = 0.0018, *h* = 0.76 mm. We measured the S_11_ characteristics using an Agilent N5230A network analyzer in an anechoic chamber to prevent interferences from external noises. Photographs of the experiment setup are in Figure 11. Five different standard dielectric MUTs from Taconic Inc. were tested. The MUTs showed relative permittivity between 2.17 and 10.2. Relative permittivity, loss tangent, and thickness [34] are summarized in Table 2.

**Figure 12:**
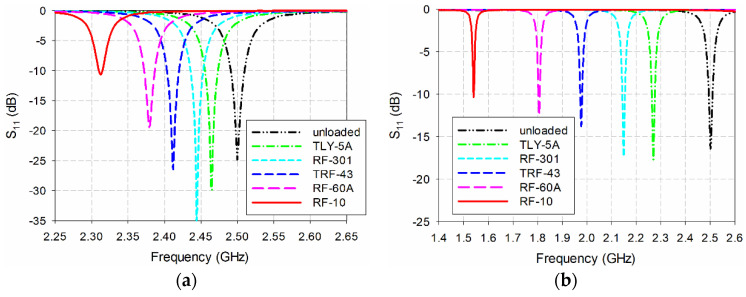
Simulated S_11_ characteristics of (**a**) the first resonant frequency of the conventional MPSA and (**b**) the second resonant frequency of the proposed scaled DR-CSRR-RA DGS-loaded MPSAs for the MUTs in Table 2.

Figure 12 shows the simulated S_11_ characteristics of the conventional and the proposed MPSAs with the five MUTs in Table 2. In order to take into account a protruding part in the SMA connector, the length of the MUTs was slightly reduced to 75 mm. Note that the MUT was placed above the patch as a superstrate for the conventional MPSA and the first resonant frequency was measured. The first resonant frequency of the conventional MPSA was used because it was used as a reference in [32,33], and the performance of the proposed MPSA can be easily compared with them. For the proposed scaled DR-CSRR-RA DGS-loaded MPSA, the MUT was placed below the ground plane, and the most sensitive second resonant frequency was measured. The first resonant frequency of the conventional MPSA shifted from 2.465 GHz for the TLY-5A MUT (*ε*_r_ = 2.17) to 2.312 GHz for the RF-10 MUT (*ε*_r_ = 10.2). For the proposed MPSA, the second resonant frequency shifted from 2.270 GHz for TLY-5A to 1.540 GHz for RF-10.

**Figure 13:**
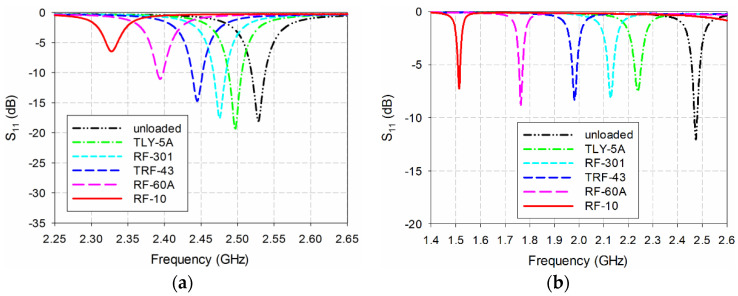
Measured S_11_ characteristics of (**a**) the first resonant frequency of the conventional MPSA and (**b**) the second resonant frequency of the proposed scaled DR-CSRR-RA DGS-loaded MPSAs for the MUTs in Table 2.

Next, the measured S_11_ characteristics of the conventional and the proposed MPSAs are in Figure 13. When unloaded, the first S_11_ resonant frequency of the conventional MPSAs was 2.528 GHz, but the second resonant frequency of the proposed MPSA was 2.474 GHz. When comparing the simulated, unloaded, first resonant frequency, the respective errors were 1.12% and 1.04%. Such errors might have been caused during fabrication and measurement including the uncertainties of the fabricated MPSAs’ substrate parameters. For instance, according to the datasheet of RF-35 [34], the relative permittivity of the fabricated RF-35 substrate can be ranged from 3.4 to 3.6, and, therefore, the error in the relative permittivity of the fabricated RF-35 substrate is ±2.86%. In order to find out the effects of the error in the relative permittivity of the fabricated RF-35 substrate on the resonant frequencies, the variations in the first resonant frequency of the conventional MPSA and the second resonant frequency of the proposed MPSA were investigated by using the simulation. It was found that the first resonant frequency of the conventional MPSA moved from 2.468 GHz (−1.28%) to 2.536 GHz (+1.44%) when the relative permittivity of RF-35 varied from 3.4 to 3.6. On the other hand, the second resonant frequency of the proposed MPSA shifted from 2.476 GHz (−0.09%) to 2.525 GHz (+1.0%). Hence, the error in the resonant frequencies of the fabricated conventional and proposed MPSAs would be within the tolerance considering the possible error in the relative permittivity of the fabricated RF-35 substrate.

For the conventional MPSA, the measured first resonant frequency moved from 2.497 GHz (TLY-5A) to 2.328 GHz (RF-10), but the second resonant frequency moved from 2.239 GHz (TLY-5A) to 1.513 GHz (RF-10) in the proposed scaled DR-CSRR-RA DGS-loaded MPSA. Table 3 summarizes simulated and measured resonant frequencies of the two MPSAs in Figure 12 and Figure 13.

**Figure 14:**
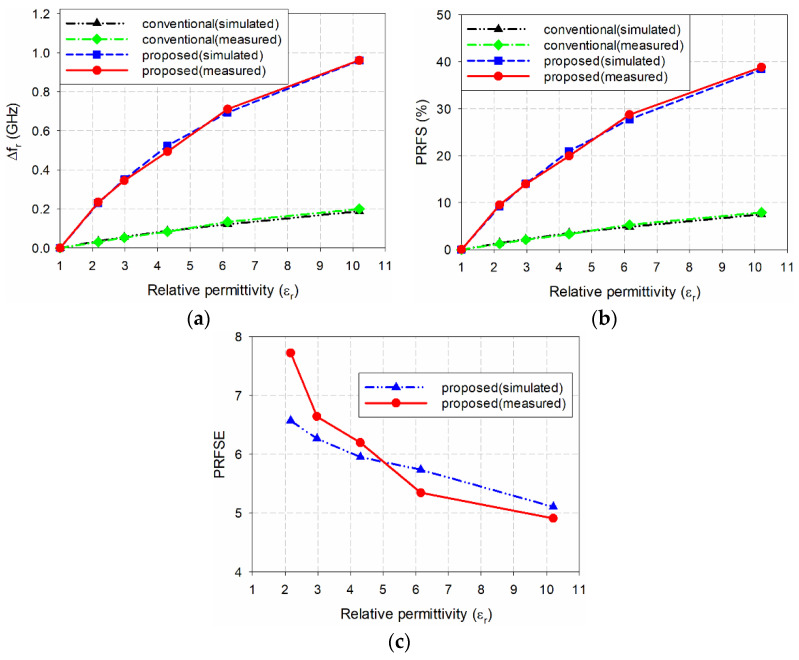
Simulated and measured performance comparison of the two MPSAs for the MUTs in Table 2: (**a**) Δ*f*_r_; (**b**) PRFS; and (**c**) PRFSE.

Figure 14 compares the simulated and measured sensitivities for the resonant frequencies. We used the TLY-5A (*ε*_r_ = 2.17), RF-301 (*ε*_r_ = 2.97), TRF-43 (*ε*_r_ = 4.3), RF-60A (*ε*_r_ = 6.15), and RF-10 (*ε*_r_ = 10.2) in turn. For the simulated results, the respective first resonant frequency shifts, Δ*f*_r_, of the conventional MPSA were 0.035, 0.056, 0.088, 0.121, and 0.188 GHz, whereas the second resonant frequency shifts of the proposed scaled DR-CSRR-RA DGS-loaded MPSA were 0.230, 0.351, 0.524, 0.694, and 0.960 GHz, respectively. PRFS for the conventional MPSA were 1.40%, 2.24%, 3.52%, 4.84%, and 7.52%, respectively, whereas PRFS for the proposed scaled DR-CSRR-RA DGS-loaded MPSA were 9.20%, 14.04%, 20.96%, 27.76%, and 38.40%, respectively. PRFSE values for the second resonant frequency of the proposed scaled DR-CSRR-RA DGS-loaded MPSA were 6.57, 6.27, 5.95, 5.74, and 5.11, respectively, compared to the first resonant frequency of the conventional MPSA. Therefore, the measured sensitivity of the second resonant frequency for the proposed MPSA was 5.11 to 6.57 times higher than the first resonant frequency of the conventional MPSA.

For measured results, the respective first resonant frequency shifts, Δ*f*_r_, of the conventional MPSA were 0.031, 0.053, 0.083, 0.134, and 0.200 GHz, whereas the second resonant frequency shifts of the proposed scaled DR-CSRR-RA DGS-loaded MPSA were 0.235, 0.345, 0.503, 0.701, and 0.961 GHz, respectively. PRFS for the conventional MPSA were 1.23%, 2.10%, 3.28%, 5.30%, and 7.91%, respectively, whereas PRFS for the proposed scaled DR-CSRR-RA DGS-loaded MPSA were 9.50%, 13.95%, 20.33%, 28.33%, and 38.84%, respectively. PRFSE values for the second resonant frequency of the proposed scaled DR-CSRR-RA DGS-loaded MPSA, compared to the first resonant frequency of the conventional MPSA, were 7.72, 6.64, 6.20, 5.35, and 4.91, respectively. Therefore, the measured sensitivity of the second resonant frequency for the proposed MPSA was 4.91 to 7.72 times higher than the first resonant frequency of the conventional MPSA. The measured results are consistent with the simulated ones with some differences. The reason for the differences might be fabrication and measurement errors including the uncertainties in the substrate parameters of the MPSAs and MUTs. The measured sensitivity of the second resonant frequency for the proposed MPSA is slightly better compared to the patch antenna loaded with a meander-line slot on the patch [33].

We validated the performance of the second resonant frequency for the proposed MPSA by extracting the relative permittivity of the five MUTs by using the measured PRFS and Equation (1). Results are in Table 4. We can see that the maximum error’s absolute value (between the extracted and the reference relative permittivity) ranged from 1.05% to 2.58%. This is within the tolerance provided by Taconic Inc. [34]. We therefore believe that the measured errors resulted from fabrication and measurement, as suggested above.

## 5. Conclusions

A high-sensitivity MPSA loaded with a DR-CSRR DGS aligned on a radiating edge of the patch was proposed in this paper. In order to find the highest sensitive resonant frequency, a comparative study of the sensitivities for the first four resonant frequencies was conducted for the conventional MPSA and for various DGS-loaded MPSAs with an RS, an SR-CSRR-OS, an SR-CSRR-IS, an R-SR-CSRR, a DR-CSRR-RA, a DR-CSRR-CA, and the proposed scaled DR-CSRR-RA. It was found that the radiating edge of the patch can be a better location to achieve higher sensitivity compared to the center of the patch, and scaling down the patch size helps to enhance the sensitivities of the higher order resonant frequencies. In addition, the sensitivities of the third and fourth resonant frequencies for the proposed scaled DR-CSRR-RA DGS-loaded MPSA were enhanced by more than two times compared to those for the DR-CSRR-RA DGS-loaded MPSA with the original patch size. The second, third, and fourth resonant frequencies of the proposed scaled DR-CSRR-RA DGS-loaded MPSA might be useful for differential sensing with similar sensitivity.

The proposed MPSA can be applied to characterize permittivity of planar solid materials and microfluidic liquid materials. Non-invasive determination of water content in soil, food, or liquids, and remote sensing of biological samples or poisonous chemicals might be other possible applications. It can also be used as a differential microwave sensor with similar sensitivity, or a multi-band microwave sensor with a single resonating element.

## Figures and Tables

**Table 1:** The first four resonant frequencies of the S_11_ responses from the eight MPSAs (in gigahertz).

MPSA Type		*ε*_r_ = 1	*ε*_r_ = 2	*ε*_r_ = 3	*ε*_r_ = 4	*ε*_r_ = 5	*ε*_r_ = 6	*ε*_r_ = 7	*ε*_r_ = 8	*ε*_r_ = 9	*ε*_r_ = 10
Conventional	*f*_r1_	2.5	2.468	2.442	2.418	2.396	2.376	2.356	2.338	2.322	2.306
	*f*_r2_	4.046	3.994	3.96	3.93	3.9	3.874	3.85	3.824	3.8	3.778
	*f*_r3_	4.766	4.712	4.666	4.626	4.59	4.556	4.522	4.492	4.462	4.434
	*f*_r4_	5.138	5.048	4.97	4.902	4.838	4.778	4.722	4.664	4.608	4.574
RS DGS	*f*_r1_	2.316	2.238	2.16	2.086	2.016	1.95	1.89	1.834	1.782	1.734
	*f*_r2_	2.75	2.684	2.644	2.618	2.6	2.588	2.58	2.572	2.566	2.56
	*f*_r3_	4.002	3.998	3.996	3.996	3.994	3.994	3.994	3.992	3.992	3.992
	*f*_r4_	4.844	4.836	4.832	4.828	4.826	4.824	4.822	4.82	4.818	4.814
SR-CSRR-OS DGS	*f*_r1_	0.768	0.752	0.738	0.724	0.712	0.7	0.688	0.678	0.668	0.658
	*f*_r2_	2.62	2.602	2.584	2.568	2.55	2.534	2.516	2.5	2.482	2.462
	*f*_r3_	3.836	3.812	3.784	3.754	3.716	3.662	3.592	3.506	3.416	3.33
	*f*_r4_	4.886	4.738	4.514	4.306	4.136	4.006	3.912	3.852	3.808	3.774
SR-CSRR-IS DGS	*f*_r1_	1.436	1.382	1.336	1.292	1.252	1.216	1.182	1.15	1.12	1.092
	*f*_r2_	2.458	2.418	2.378	2.342	2.304	2.268	2.232	2.198	2.166	2.134
	*f*_r3_	3.406	3.21	3.082	2.988	2.916	2.858	2.81	2.772	2.738	2.71
	*f*_r4_	4.152	4.14	4.128	4.118	4.106	4.094	4.082	4.07	4.058	4.044
R-SR-CSRR DGS	*f*_r1_	1.638	1.62	1.602	1.586	1.568	1.552	1.536	1.52	1.504	1.488
	*f*_r2_	2.968	2.85	2.692	2.546	2.42	2.31	2.214	2.128	2.052	1.984
	*f*_r3_	3.28	3.122	3.056	3.02	2.994	2.972	2.95	2.932	2.914	2.898
	*f*_r4_	3.918	3.904	3.892	3.882	3.87	3.86	3.85	3.838	3.828	3.816
DR-CSRR-RA DGS	*f*_r1_	0.64	0.626	0.614	0.602	0.592	0.582	0.572	0.562	0.554	0.546
	*f*_r2_	2.266	2.096	1.958	1.842	1.744	1.662	1.588	1.524	1.468	1.416
	*f*_r3_	2.638	2.596	2.57	2.546	2.524	2.502	2.48	2.458	2.434	2.41
	*f*_r4_	3.87	3.85	3.84	3.802	3.754	3.688	3.592	3.486	3.386	3.284
DR-CSRR-CA DGS	*f*_r1_	0.644	0.632	0.622	0.612	0.604	0.594	0.586	0.58	0.572	0.564
	*f*_r2_	1.896	1.844	1.788	1.732	1.678	1.624	1.574	1.528	1.486	1.446
	*f*_r3_	2.498	2.398	2.326	2.276	2.238	2.208	2.184	2.166	2.15	2.134
	*f*_r4_	3.746	3.684	3.618	3.546	3.47	3.39	3.308	3.23	3.152	3.078
Proposed	*f*_r1_	0.808	0.783	0.764	0.747	0.728	0.712	0.695	0.681	0.672	0.658
	*f*_r2_	2.5	2.302	2.142	2.014	1.906	1.814	1.734	1.664	1.602	1.546
	*f*_r3_	4.09	3.86	3.676	3.516	3.374	3.248	3.138	3.04	2.952	2.872
	*f*_r4_	5.318	4.966	4.682	4.444	4.238	4.06	3.902	3.762	3.636	3.522

**Table 2:** Relative permittivity, loss tangent, and thickness of the five MUTs.

No.	MUT	*ε*_r_	tan δ	Thickness
1	TLY-5A	2.17 ± 0.02	0.0009@10GHz	1.58 mm
2	RF-301	2.97 ± 0.07	0.0012@1.9GHz	1.52 mm
3	TRF-43	4.3 ± 0.15	0.0035@10GHz	1.63 mm
4	RF-60A	6.15 ± 0.15	0.0028@10GHz	1.52 mm
5	RF-10	10.2 ± 0.3	0.0025@10GHz	1.52 mm

**Table 3:** Simulated and measured resonant frequencies of S_11_ responses for the two MPSAs (in gigahertz).

MPSA Type	Unloaded (*ε*_r_ = 1)	TLY-5A (*ε*_r_ = 2.17)	RF-301 (*ε*_r_ = 2.97)	TRF-43 (*ε*_r_ = 4.3)	RF-60A (*ε*_r_ = 6.15)	RF-10 (*ε*_r_ = 10.2)
Conventional (*f*_r1_, simulated)	2.5	2.465	2.444	2.412	2.379	2.312
Conventional (*f*_r1_, measured)	2.528	2.497	2.475	2.445	2.394	2.328
Proposed (*f*_r2_, simulated)	2.5	2.270	2.149	1.976	1.806	1.540
Proposed (*f*_r2_, measured)	2.474	2.239	2.129	1.971	1.773	1.513

**Table 4:** Comparison of extracted relative permittivity for the five MUTs.

No.	MUT	Reference *ε*_r_	Extracted *ε*_r_	Error (%)
1	TLY-5A	2.17 ± 0.02	2.2261	−2.58
2	RF-301	2.97 ± 0.07	2.9388	1.05
3	TRF-43	4.3 ± 0.15	4.1899	2.56
4	RF-60A	6.15 ± 0.15	6.2655	−1.88
5	RF-10	10.2 ± 0.3	10.3290	−1.27

## References

[1] Saeed K., Shafique M.F., Byrne M.B., Hunter I.C., Haq M.Z. (2012). Planar microwave sensors for complex permittivity characterization of materials and their applications. Applied Measurement System.

[2] Baker-Jarvis J., Vanzura E., Kissick W. (1990). Improved technique for determining complex permittivity with the transmission/reflection method. IEEE Trans. Microw. Theory Tech..

[3] Varadan V.V., Hollinger R., Ghodgaonkar D., Varadan V.K. (1991). Free-space, broadband measurements of high-temperature, complex dielectric properties at microwave frequencies. IEEE Trans. Instrum. Meas..

[4] Grant J.P., Clarke R.N., Symm G.T., Spyrou N.M. (1989). A critical study of the open-ended coaxial line sensor for RF and microwave complex permittivity measurements. J. Phys. E Sci. Instrum..

[5] Raj A., Holmes W., Judah S. (2001). Wide bandwidth measurement of complex permittivity of liquids using coplanar lines. IEEE Trans. Instrum. Meas..

[6] Mathew K.T., Raveendranath U. (1993). Waveguide cavity perturbation method for measuring complex permittivity of water. Microw. Opt. Technol. Lett..

[7] Raveendranath U., Bijukumar S., Matthew K. (2000). Broadband coaxial cavity resonator for complex permittivity measurements of liquids. IEEE Trans. Instrum. Meas..

[8] Boybay M.S., Ramahi O.M. (2012). Material characterization using complementary split-ring resonators. IEEE Trans. Instrum. Meas..

[9] Withayachumnankul W., Jaruwongrungsee K., Adisorn T., Fumeaux C., Abbott D. (2013). Metamaterial-based microfluidic sensor for dielectric characterization. Sens. Actuators A-Phys..

[10] Chen T., Li S., Sun H. (2012). Metamaterials application in sensing. Sensors.

[11] KT M.S., Ansari M.A.H., Jha A.K., Akhtar M.J. (2017). Design of SRR-based microwave sensor for characterization of magnetodielectric substrates. IEEE Microw. Wirel. Compon. Lett..

[12] Sha K.T.M., Jha A.K., Akhtar M.J. (2017). Improved planar resonant RF sensor for retrieval of permittivity and permeability of materials. IEEE Sens. J..

[13] Velez P., Su L., Grenier K., Mata-Contreras J., Dubuc D., Martin F. (2017). Microwave microfluidic sensor based on a microstrip splitter/combiner configuration and split ring Resonators (SRRs) for dielectric characterization of liquids. IEEE Sens. J..

[14] Ebrahimi A., Scott J., Ghorbani K. (2018). Differential sensors using microstrip lines loaded with two split ring resonators. IEEE Sens. J..

[15] Lee C.S., Yang C.L. (2014). Thickness and permittivity measurement in multi-layered dielectric structures using complementary split-ring resonators. IEEE Sens. J..

[16] Lee C.S., Yang C.L. (2014). Complementary split-ring resonators for measuring dielectric constants and loss tangents. IEEE Microw. Wirel. Compon. Lett..

[17] Ebrahimi A., Withayachumnankul W., Al-Sarawi S., Abbott D. (2014). High-sensitivity metamaterial-inspired sensor for microfluidic dielectric characterization. IEEE Sens. J..

[18] Ansari M.A.H., Jha A.K., Akhtar M.J. (2015). Design and application of the CSRR-based planar sensor for noninvasive measurement of complex permittivity. IEEE Sens. J..

[19] Yang C.L., Lee C.S., Chen K.W., Chen K.Z. (2016). Noncontact measurement of complex permittivity and thickness by using planar resonators. IEEE Trans. Microw. Theory Tech..

[20] Su L., Mata-Contreras J., Naqui J., Martín F. (2016). Splitter/combiner microstrip sections loaded with pairs of complementary split ring resonators (CSRRs): Modeling and optimization for differential sensing applications. IEEE Trans. Microw. Theory Tech..

[21] Su L., Mata-Contreras J., Vélez P., Fernández-Prieto A. (2018). Analytical method to estimate the complex permittivity of oil samples. Sensors.

[22] Ansari M.A.H., Jha A.K., Akhter Z., Akhtar M.J. (2018). Multi-band RF planar sensor using complementary split ring resonator for testing of dielectric materials. IEEE Sens. J..

[23] Saadat-Safa M., Nayyeri V., Khanjarian M., Soleimani M., Ramahi O.M. (2019). A CSRR-based sensor for full characterization of magneto-dielectric materials. IEEE Trans. Microw. Theory Tech..

[24] Yeo J., Lee J.-I. (2019). High-sensitivity microwave sensor based on an interdigital-capacitor-shaped defected ground structure for permittivity characterization. Sensors.

[25] Guha D., Biswas S., Antar Y.M.M. (2011). Defected ground structure for microstrip Antennas. Microstrip and Printed Antennas: New Trends, Techniques and Applications.

[26] Kumar A., Machavaram K.V. (2013). Microstrip filter with defected ground structure: A close perspective. Int. J. Microw. Wirel. Technol..

[27] Khandelwal M.K., Kanaujia B.K., Kumar S. (2017). Defected ground structure: Fundamentals, analysis, and applications in modern wireless trends. Int. J. Antennas Propag..

[28] Xie Y.H., Zhu C., Li L., Liang C.H. (2012). A novel dual-band metamaterials antenna based on complementary split ring resonators. Microw. Opt. Technol. Lett..

[29] Pandeeswari R., Raghavan S. (2015). Microstrip antenna with complementary split ring resonator loaded ground plane for gain enhancement. Microw. Opt. Technol. Lett..

[30] Raval F., Kosta Y., Joshi H. (2015). Reduced size patch antenna using complementary split ring resonator as defected ground plane. AEU-Int. J. Electron. Commun..

[31] Huang Y., Boyle K. (2008). Antennas: From theory to Practice.

[32] Yeo J., Lee J.-I. (2019). Slot-loaded microstrip patch sensor antenna for high-sensitivity permittivity characterization. Electronics.

[33] Yeo J., Lee J.-I. (2019). Meander-line slot-loaded high-sensitivity microstrip patch sensor antenna for relative permittivity measurement. Sensors.

[34] Taconic PTFE Laminates. http://www.taconic.co.kr/pages/sub02_03.php.

